# Soft robotic devices for hand rehabilitation and assistance: a narrative review

**DOI:** 10.1186/s12984-018-0350-6

**Published:** 2018-02-17

**Authors:** Chia-Ye Chu, Rita M. Patterson

**Affiliations:** 1 Texas College of Osteopathic Medicine, University of North Texas Health Science Center, 3500 Camp Bowie Blvd, Fort Worth, 76107 TX USA; 2 Department of Family and Manipulative Medicine, University of North Texas Health Science Center, 3500 Camp Bowie Blvd, Fort Worth, 76107 TX USA

**Keywords:** Soft robotics, Wearable robots, Rehabilitation, Hand

## Abstract

**Introduction:**

The debilitating effects on hand function from a number of a neurologic disorders has given rise to the development of rehabilitative robotic devices aimed at restoring hand function in these patients. To combat the shortcomings of previous traditional robotics, soft robotics are rapidly emerging as an alternative due to their inherent safety, less complex designs, and increased potential for portability and efficacy. While several groups have begun designing devices, there are few devices that have progressed enough to provide clinical evidence of their design’s therapeutic abilities. Therefore, a global review of devices that have been previously attempted could facilitate the development of new and improved devices in the next step towards obtaining clinical proof of the rehabilitative effects of soft robotics in hand dysfunction.

**Methods:**

A literature search was performed in SportDiscus, Pubmed, Scopus, and Web of Science for articles related to the design of soft robotic devices for hand rehabilitation. A framework of the key design elements of the devices was developed to ease the comparison of the various approaches to building them. This framework includes an analysis of the trends in portability, safety features, user intent detection methods, actuation systems, total DOF, number of independent actuators, device weight, evaluation metrics, and modes of rehabilitation.

**Results:**

In this study, a total of 62 articles representing 44 unique devices were identified and summarized according to the framework we developed to compare different design aspects. By far, the most common type of device was that which used a pneumatic actuator to guide finger flexion/extension. However, the remainder of our framework elements yielded more heterogeneous results. Consequently, those results are summarized and the advantages and disadvantages of many design choices as well as their rationales were highlighted.

**Conclusion:**

The past 3 years has seen a rapid increase in the development of soft robotic devices for hand rehabilitative applications. These mostly preclinical research prototypes display a wide range of technical solutions which have been highlighted in the framework developed in this analysis. More work needs to be done in actuator design, safety, and implementation in order for these devices to progress to clinical trials. It is our goal that this review will guide future developers through the various design considerations in order to develop better devices for patients with hand impairments.

## Background

Imagine tying your shoes or putting on a pair of pants while having limited use of your hands. Now imagine the impact on your daily life if that limitation was permanent. The ability to perform activities of daily living (ADL) is highly dependent on hand function, leaving those suffering with hand impairments less capable of executing ADLs and with a reduced quality of life. Unfortunately, the hand is often the last part of the body to receive rehabilitation.

According to a 2015 National Health Interview Survey, there were approximately 4.7 million adults in the United States that found it “Very difficult to or cannot grasp or handle small objects” [1]. Hand impairments are commonly observed in neurological and musculoskeletal diseases such as arthritis, Cerebral Palsy, Parkinson’s Disease, and stroke. A summary of motor impairment prevalence associated with these diseases may be seen in Table 1. Fortunately, physical rehabilitation has been shown to promote motor recovery through repetitive isolated movements [2–5]. This is largely due to neuroplasticity – the ability for the brain to reorganize itself by establishing new neural connections. Occupational and physical therapists thus attempt to take advantage of neuroplasticity in order to re-map motor function in the brain through repeated exercise. Currently, however, there is no consensus on the best mode and dosing to facilitate neuroplasticity [6]. Additionally, recovery success relies heavily on a patient’s ability to attend therapy, which can be deterred by the frequency, duration, or cost of the therapy. Robotic devices could enhance access to repeated exercise. As such, they have been developed and investigated for their utilization as an adjunctive therapy to improve patient access, compliance and subsequent outcomes of rehabilitation efforts. An overview of the designs with comparisons between the different approaches will help future development of these tools.

**Table 1:** Common disorders and upper extremity motor impairment prevalence

Disease	Disease Prevalence (US cases per year)	Motor Impairment Prevalence*	Type of Upper Extremity Impairment
Arthritis [21]	78 million (projected prevalence by 2040)	3 million (2009)	Grasping
Cerebral palsy [22]	1 in 323 children (2008)	~ 50% of children	Arm-hand dysfunction
Parkinson’s Disease [23]	500,000 (2010)	Not reported	Tremor, rigidity, akinesia/bradykinesia
Spinal Cord Injury [24]	282,000 (2016)	58.3%	Tetraplegia
Stroke [25, 26]	795,000 (incidence, 2016)	50%	Upper extremity hemiplegia

^*^Motor impairment prevalence values correspond only to the specific impairments listed under Type of Upper Extremity Impairment (other motor impairments may be seen within these diseases)

It is thought that the benefits of a robotic device for rehabilitation purposes include: more intense and longer therapy sessions, feedback mechanisms to amplify movements, automated sessions to reduce therapy hours, automation of patient-specific therapy based on degree of motor impairment, and more precise measurements of motor function [7]. Despite these advantages, a comprehensive review by Maciejasz et al. of over 120 robotic rehabilitation devices for the upper limb failed to locate sufficient clinical evidence proving their efficacy over conventional therapies [8]. Their review also included a meta-analysis of trials of stroke patients undergoing robotic training, which suggested that while motor impairment was improved, the ability to perform ADLs was not. This lack of clinical evidence could be attributable to the hard materials – mostly metals – that conventional robotics are composed of and which provide a rigid framework in order to assist in motor function. It is possible that the rigid structures of these devices is impeding the therapeutic potential of robotics by reducing their biomimetic qualities. This may include reducing motion in unactuated directions such as finger abduction or could include having rigid axes of rotation that become misaligned with the finger’s anatomic axis during motion.

In contrast, soft robotics are fabricated from easily deformable materials such as fluids, gels, and soft polymers that have better biomimetic qualities due to their increased compliance and versatility while conforming to the contours of the human body. The lack of rigid components removes constraints on non-actuated degrees of freedom and also reduces joint alignment issues, which could prevent joint damage [9]. Additionally, soft robotics may be lighter and have simpler designs, making them more likely to be portable and opening up the possibility of at-home rehabilitation. This would allow patients to train in the comfort of their own home, possibly reducing overall rehabilitation costs. Home rehabilitation could also increase patient compliance, leading to more active therapy sessions and hopefully improved outcomes.

Soft robotic devices have been developed for rehabilitative applications for most major joints of the body, including the ankle, knees, shoulder, elbows, and wrists [10–14]. There have been many attempts to design these soft robotic devices for hand rehabilitation applications, but there is little clinical evidence to support any particular design solution over another. Therefore, a detailed review of devices will be useful for future developers by guiding them through the successes and shortcomings of previous designs. This paper aims to facilitate the development of soft robotic devices for hand rehabilitation by reviewing previous designs of soft robotic devices for the hand.

## Methods

### Search strategy

This narrative review was conducted by performing a literature search in SportDiscus, Pubmed, Scopus, and Web of Science using their respective controlled vocabularies for terms related to soft robotics and rehabilitation of the hand. Appropriate syntax using Boolean operators and wildcard symbols was used for each database to include a wider range of articles that may have used alternate spelling or synonyms (Table 2).

**Table 2:** Databases used and respective search entries

Database	Search query
SportDiscus	TX “soft robot^*^”
Pubmed	(“soft robot^*^” [All Fields] OR (“Robotics” [MeSH] AND “soft” [All Fields])) AND (hand OR finger OR thumb OR glove [All fields])
Scopus	TITLE-ABS-KEY(Robot^*^) AND TITLE-ABS-KEY(Soft) AND TITLE-ABS-KEY(“hand” OR “finger” OR “thumb” OR “glove”)
Web of Science	TI = (soft robot^*^) AND TI = (hand OR finger OR thumb OR glove)

*Denotes a search entity for Robot

After obtaining primary search results from each database, the following inclusion and exclusion criteria were used to further narrow the literature search:

Inclusion criteria:

- The device described was a soft robot: No rigid components on the robot-human interface or minimal rigid components that will not impose physical restraints on joint motions
- The device intended to facilitate the movement of at least one finger joint in the hand
- The paper was a scientific article written in the English language and accessible to the authors

Exclusion criteria:

- The device contained rigid components on the robot-human interface that could reasonably impose physical restraints on joint motions
- The device focused on other joints without including the fingers
- There was insufficient information about the device’s design such that analysis was not clear
- The device was intended for use as a prosthetic

While there are a number of devices aimed at rehabilitation of other major joints of the body, they were not included in this review because the kinematics of the hand are complex and as such the design considerations are more unique. Additionally, prosthetic devices which replace human anatomy were deemed to have design considerations that differ from the focus on rehabilitation and so were similarly excluded. Finally, after narrowing our search results, the reference sections of each article were also screened for any devices that fit the search criteria but were not identified previously. These references were the final source of devices for this review.

### Framework for comparison

**Fig. 1:**
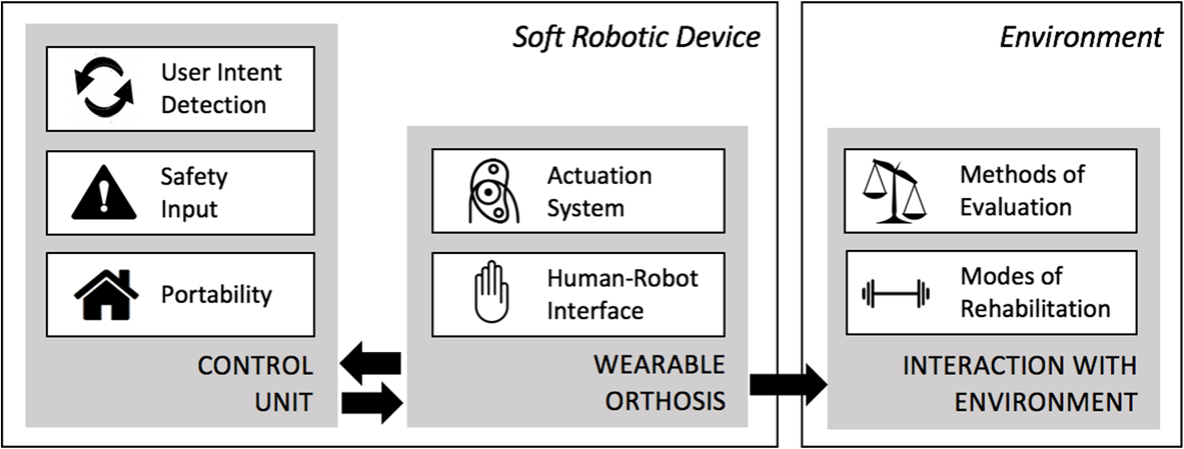
Soft robotic major components schematic

In order to guide the analysis of a relatively large number of devices, a general framework was developed (Fig. 1) that conceptualizes the essential components of each device and the basic interactions between these components. Even though in reality, the precise interactions between the various components are much more complex than depicted, not all interactions are immediately critical for the analysis, and may hinder the clarity of comparisons. Instead, the schematic depicting major design components allows easy comparison between the various technical solutions to designing these robots, which will provide insight into the current state of development and help guide future work in the field. Our discussion of the schematic is broken into two parts: the first part deals with the design of the robotic device while the second part deals with how the device interacts with the environment.

#### Soft robotic device

The first part of the framework which deals with the design of these devices is further broken down into two basic functional units: the *Control Unit* and the *Wearable Orthosis*. Beginning with the *Control Unit*, there are many design considerations that go into developing this part of the device and they can be quite complicated with subtleties that are difficult to summarize across many devices. Therefore, the analysis of the *Control Unit* focuses on a few essential characteristics which will help these devices advance to the next steps in development. These characteristics include portability, safety mechanisms, and user intent detection modalities. To further clarify, portability in this context is defined as the ability for the device to be used in a patient’s home without the aid of a clinician or technician. This is because home rehabilitation has been shown to be beneficial for functional and psychological performance [15] and would ideally lead to shorter yet more effective therapy regimes. Next, the safety mechanism, which is often in the form of some feedback signal that tells the *Control Unit* to power down the device, is important for these robots in order to provide the patient with some form of protection should the device begin to malfunction. Finally, the user intent detection modality is any signal that monitors the patient’s attempt to move their hand and is sent to the device’s *Control Unit* to augment that movement. Not only does this feedback control facilitate neuroplasticity in the rehabilitation process, but it can also allow for objective evaluation of robot motion and clinical outcome recovery. Thus, a comparison between the different modalities could drive future rehabilitation strategies.

The other half of this framework concerned with device design is the *Wearable Orthosis*, which is considered the minimal equipment that must be in contact with the hand in order to operate. This part of the discussion was divided into two related parts: the actuation system and the human-robot interface. The actuation system is defined as the mechanical system in place that is used to guide finger joint movement. The various approaches to designing these systems have inherent advantages and disadvantages that need to be understood as they directly influence the human-robot interface. This interface will cover each device’s total degrees of freedom (DOF), number of independently controlled actuators, and weight of the hand orthosis. For these devices, the DOF will be counted as 1 DOF for flexion/extension of each of the distal interphalangeal joints, proximal interphalangeal joints, and metacarpophalangeal joints. The thumb will be counted as 1 DOF each for flexion/extension of the interphalangeal joint, metacarpophalangeal joint, and carpometacarpal joint. Abduction/adduction of joints will also be counted when mentioned in their design. The weight of the device will include only the portion of the device that is worn on the hand and should weigh less than 0.5 kg [16] in order to reduce any unnecessary weight that could hinder movement of the impaired patient’s hand.

#### Environment

The final component of this framework is focused on these devices’ *Interaction with the Environment*. The first half of this section focuses on the different metrics that have been used to evaluate these devices. All of these devices are uniquely built and have been evaluated in many ways, but there is no real standard by which to compare them. Therefore, an analysis of the studies that have been completed can help guide future developers in their experiments by understanding the most commonly used metrics. This would facilitate an ease of comparison between these devices and provide a way to monitor the progression of these devices. The second half of this section is dedicated to observing the trends in the types of rehabilitation these devices intend to implement. This includes Active Resistance (AR), Continuous Passive Motion (CPM), Task Specific Training (TST), and Virtual Reality (VR). A glossary of these terms can be seen in Table 3. It is assumed that all of these devices can provide active assistance (assistance as the patient attempts to engage hand movement) and passive assistance (assistance as the patient remains idle) so these were not specifically included. An analysis into this trend among devices will help shed light onto the rationales for choosing different training modalities in rehabilitative applications.

**Table 3:** Glossary of terms for modes of rehabilitation

Mode	Description
Active Resistance (AR)	Patient attempts to exercise hand against a resistive force from the device
Continuous Passive Motion (CPM)	Patient is subjected to repetitive motion by the device
Task Specific Training (TST)	Patient is given a specific action to complete (ie grabbing a ball) while the device provides assistance
Virtual Reality (VR)	Patient is placed in a virtual reality while the device assists in various activities

While this framework does not address the exact technology to build a soft robotic device, its utility lies in its compartmentalization of various aspects of device design which facilitates an ease of comparison between the different approaches to building these devices.

## Results: literature collection and framework summary overview

### Literature collection

**Fig. 2:**
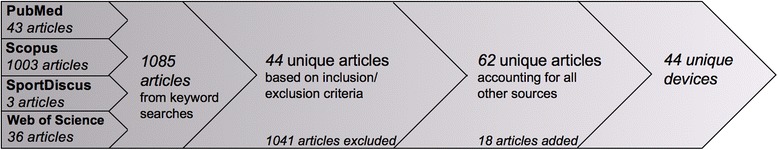
Literature search process and results

The final search queries for this review were completed on November 10th, 2017. A total of 62 articles, representing 44 unique devices were identified for this overview. A summary of the article selection procedure can be seen in Fig. 2.

### Overview summary of component development

A summary of all the devices used for this analysis, grouped according to their type of actuation, is shown in Table 4. Also included in the table are the results of the analysis of each individual device according to the aforementioned framework. For work groups that had multiple publications, devices were analyzed separately if they were clearly different designs. Those that were improvements upon previous designs were grouped together and the most recent iteration of the device was used for this analysis.

**Table 4:** Summary of framework analysis

Device / Group	Assisted Motion	Portability	Safety	User Intent Modality	Total DOF	No. Ind. Actuators	Weight (g)	Input force	Ext. torque / Grip force
Cable systems									
Biggar et al. [27]	F	Y	–	–	9	3	–		
Cao et al. [28]	F	–	–	sEMG	9	1	50		
Exo-Glove Poly / Kang et al. [29]	E/F	Y	–	–	9	2	–		- / 29.5 N
Exo-Glove / In et al. [30–32]	E/F	Y	–	Bend sensors	9	3	194	50 N	- / 40 N
GraspyGlove / Popov et al. [33]	E/F	Y	–	–	12	1	250		
GRIPIT / Kim et al. [34]	F	–	–	–	9	1	40		
IronHand / Radder et al. [35–37]	F	Y	–	Pressure sensors	9	–	70		
Nycz et al. [38, 39]	E/F	Y	Spool rotational limit	sEMG	15	1	–		
Park et al. [40]	E/F	Y	Magnetic coupling	–	15	2	–	34 N	- / 35 N
RoboGlove / Diftler et al. [17]	F	Y	Multi-modal	–	15	3	771		- / 222 N
SEM Glove / Nilsson et al. [41]	F	Y	–	Pressure sensors	9	3	–	20 N	- / 24 N
VAEDA Glove / Theilbar et al. [42–45]	E	Y	Verbal command	sEMG + Voice	15	1	225		
Xiloyannis et al. [46]	F	Y	–	–	9	1	–		
Yao et al. [47]	E/F	–	–	–	17	–	85		- / 11 N
Yi et al. [48]	E/F	–	–	–	12	–	< 100		
Pneumatic systems									
Al-Fahaam et al. [49]	F	Y	Pinky control	–	12	–	100	400 kPa	- / 17 N
Coffey et al. [50]	E	Y	–	EEG	15	1	–		
Exo-Glove PM / Yun et al. [51]	F	Y	Pressure sensor	–	16	1	–	300 kPa	- / 22 N
Kline et al. [19]	E	–	Pressure sensor	sEMG	15	1	100	34 kPa	< 1 Nm / -
Li et al. [52]	E	–	–	–	15	1	–		
Low et al. [53]	F	–	–	–	3	1	25		
Maeder-York et al. [54]	F	Y	–	–	3	1	–	207 kPa	
MR Glove / Yap et al. [12, 55]	F	Y	–	–	12	–	180	120 kPa	- / 41 N
Nordin et al. [56]	F	–	Emergency button	–	15	3	–	200 kPa	- / 3.61 N
Noritsugu et al. [57]	F	–	–	–	15	2	120	500 kPa	
PneuGlove / Connelly et al. [20]	E	–	Bend sensor	–	15	5		68.9 kPa	2.7 Nm / -
Polygerinos et al. [58]	F	–	–	–	12	1	160	43 kPa	- / 4.42 N
Power Assist Glove / Toya et al. [18]	F	–	–	Bend sensors	15	4	180		
PowerAssist Glove / Kadowaki et al. [59]	E/F	–	–	sEMG	15	–	135		
RARD / Chua et al. [60]	Abduction/Adduction	–	–	–	1	1	–		
REHAB Glove / Hagshenas-Jaryani et al. [61–64]	F	–	Pressure sensor	–	15	5	–	50 kPa	
Reymundo et al. [65]	E	–	–	–	3	1	–	50 kPa	
Tarvainen et al. [66]	F	–	–	–	3	2	–		
Wang et al. [67]	E/F	–	–	–	15	5	–	675 kPa	- / 21.24 N
Wang et al. [68]	F	–	–	–	3	1	–	350 kPa	
Yap et al. [69, 70]	F	Y	–	sEMG	15	5	170	120 kPa	- / 6.5 N
Yap et al. [15, 71]	F	–	–	–	12	1	200		
Yap et al. [72, 73]	E/F	Y	–	–	15	5	180	120 kPa	- / 8.4 N
Yap et al. [74]	E	Y	–	–	15	5	150	100 kPa	4.25 Nm / -
Yap et al. [75]	F	–	–	–	3	1	–	200 kPa	
Yeo et al. [76]	F	–	Strain sensor	–	3	1	–	110 kPa	
Zaid et al. [77]	F	–	–	–	6	2	–		
Zhang et al. [78]	F	–	–	–	3	1	–		
Hydraulic systems									
Polygerinos et al. [22, 79, 80]	F	Y	Emergency button	sEMG	15	5	285	413 kPa	- / 14.15 N

*E* extension, F flexion, *‘-‘*value not reported

**Fig. 3:**
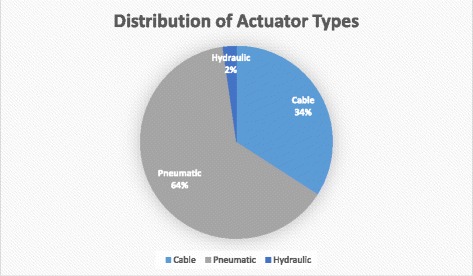
Distribution of actuator types

The data are grouped according to their type of actuation in order to ease their comparison. The types of actuators identified include: Cable systems, Pneumatic systems, and Hydraulic systems. A distribution of the prevalence of each type of actuator is seen in Fig. 3. Cable systems were those that used cables to attach at the distal phalanx and can guide finger flexion or extension when tension is applied. This was designed to mimic the tendon system of the flexor and extensor muscles of the hand. Pneumatic systems were those that used pressurized air through an actuator in contact with the hand to cause finger flexion and/or extension. Hydraulic systems were similar but used some type of fluid instead of air to pressurize the actuator.

## Discussion of framework elements

### Soft robotic device design

#### Portability

Twenty out of the 44 (45%) devices analyzed were designed with portability in mind. Of these portable devices, 85% were published in the last 3 years which shows that this trend towards home rehabilitation is a recent one. Given the multitude of benefits potentially attainable through home rehabilitation, we expect that there will be an increasing trend towards developing devices that are fully capable of being operated in a patient’s own home without the need of a clinician or technician. In addition to portability, several devices were observed to be mobile due to compact control units that were battery powered. To clarify, a portable device was not necessarily mobile since some portable devices were powered by wall outlets but could still be used in a patient’s home. Mobility enabled users to power the device while freely moving about their environment, which permitted more assistance with their ADL’s compared to if they were tethered to a wall outlet. This allowed devices to have further applications as assistive devices. Currently, however, it is unclear what the benefit having this mobility grants in terms of rehabilitative success and so it should be elucidated to determine whether it is a desirable trait in these rehabilitative devices. On the other hand, it is much clearer how portability could influence outcomes and should be strived for in as many devices as possible for future evaluations to validate those claims.

#### Safety input

In terms of safety measures, only 12 of the 44 devices (27%) had implemented clear forms of safety mechanisms, which was lower than expected given the importance of safety in robotics. Additional devices may have safety precautions built into their device’s control algorithms, but the details of those algorithms were beyond the scope of this analysis. Instead, this discussion focuses on those additional safety measures that have been implemented and reported in the literature. A description of each safety mechanism as well as their foreseeable advantages and disadvantages are shown in Table 5. It is likely that this lack of safety provisions is also due to the premature stages of many devices, which few have tested on human subjects and so safety is not yet a major concern within their designs. However, a brief discussion of the current methods may still help guide the future development of these devices. A general trend that occurred among all devices was a desire to reduce the components on the wearable orthosis. By incorporating the safety mechanisms into the control units, any additional weight could be kept off of the patient’s impaired hand which is ideal for the rehabilitative process. For example, consider pneumatic actuators that monitor pressure values which correspond to a certain degree of bending. While inferring safety from regulating secondary metrics like pressure is easy to implement, it should be cautioned that patient variability should be accounted for and may affect the safety metric. For example, a severely impaired patient with very stiff joints may have a higher pressure threshold compared to a mildly impaired patient. On the other hand, a number of devices were able to directly measure joint bending, such as through bend sensors or strain sensors, and use that information to regulate actuation of the digits. As long as the inclusion of these sensors does not add significant weight on the hand or impede the motions of the digits, this may be an effective method of monitoring these devices to prevent harm to patients. Additionally, in terms of safety considerations, only one device had multiple forms of feedback modalities [17]. This device not only accounted for the degree of actuation but also for electrical malfunction of the device. While this device was not originally designed for rehabilitative purposes, this multi-modal form of safety would be beneficial for rehabilitative robotic devices. In summary, inclusion of effective safety mechanisms are an absolute necessity for future iterations of these devices.

**Table 5:** Description of safety mechanisms with relative advantages and disadvantages

Safety Mechanism	Description	Advantages	Disadvantages
Spool rotational limit	Spools which guide the cables are limited in rotation, thereby preventing hyper-flexion/ extension	Intrinsically built into the system to avoid over-actuation of the digits.	Possibility of failure if patient initiates device mode incorrectly
Pressure sensor	Pressure sensor shuts off actuation when threshold pressure is exceeded	Sensor can be easily incorporated into control unit	Pressure thresholds may not be the same among patients with differing degrees of impairment
Emergency button	A button is available on the control unit to provide immediate cessation of actuation	Patient has ability to override the device when they sense discomfort	Patients with impairments may not react quickly to prevent damage from severe malfunction
Bend sensors / Strain sensors	Sensors placed along the joints can detect and control the degree of bending	Can more directly measure the degree of joint bending	May be more difficult to implement and must be cautious when adding them to the hand orthosis
Unactuated digit detection	Monitors the movement of a digit that is not actuated so that the patient’s voluntary movement of that digit sends a signal to the device to turn off	Patient has ability to determine when to shut off the device	Requires residual function in a digit, forces device to leave at least one digit unactuated
Magnetic coupling	The actuator cables are magnetically coupled to the robotic tendons and detach when the tension is too high	Patient does not need to alert for termination	May be difficult to customize for varying levels of hand dysfunction
Verbal command	The user says a verbal command, such as “stop”	Patient can quickly terminate device	Voice recognition failure
Multi-modal feedback	Sensors for temperature, motor current, battery levels, and loss of sensor feedback all have the ability to cease operation	Many layers of security to greater ensure protection from electrical components of device	Only motor current to the actuators is vaguely correlated to degree of finger movement

#### Feedback input

**Fig. 4:**
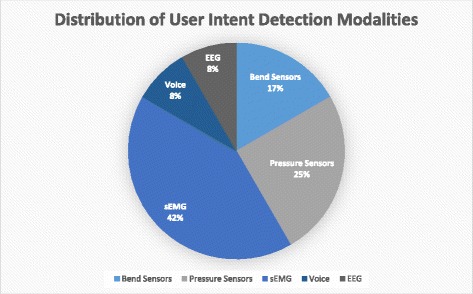
Distribution of feedback modalities

**Fig. 5:**
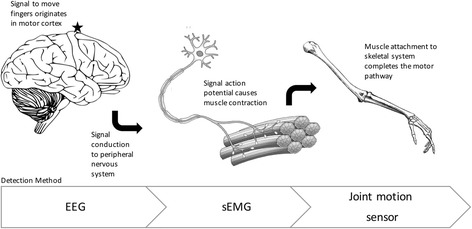
Methods of detection along motor pathway [81]

The ability to detect a user’s intent to engage hand movement is a major advantage these robotic devices have over traditional rehabilitation yet currently, only about 30% (13/44) of the devices reviewed reported this functionality. A distribution of the feedback modalities that have been investigated is shown in Fig. 4. Some devices were developed primarily as assistive devices and so detecting user intent was not necessarily a priority, which may help explain why there were relatively few devices capable of detecting intent. Nevertheless, this section will focus on the current methods that have been attempted. These include bend and pressure sensors on the digits or wrist, electroencephalography (EEG) readings, surface electromyography (sEMG) readings, and voice activation. A description of these modalities as well as their relative advantages and disadvantages may be seen in Table 6. The major difference between these modalities is the location along the motor pathway that they detect intent (Fig. 5). Detecting user intent near the end of the motor pathway, such as by measuring joint movement, is easy to implement and acquire a reliable signal. However, it requires that the patients have some residual function and requires additional hardware around the hand that may affect the weight or dynamics of the device. On the other hand, signal detection at the beginning of the pathway through EEG may extend application to patients with complete paralysis but is much more complex to implement due to the numerous electrodes and intensive signal processing required. Instead, sEMG was a compromise in terms of reliable signal acquisition and ease of implementation and explains why it was the most widely used method of detecting user intent (50% of devices with feedback). One device combined sEMG with voice recognition in order to further enhance detecting intent, making it the only device noted to have any multi-modal approach. This could open the possibility of other combinations that can further enhance the rehabilitation process. Regardless, it appears that there is a slower trend towards incorporating at least one feedback modality to detect user intent accompanied by robotic augmentation of that intent.

**Table 6:** Description of user intent detection modalities with relative advantages and disadvantages

Feedback Modality	Description	Advantages	Disadvantages
Bend Sensors – Digits	Sensors are placed on all finger joints and a joint pattern analysis can detect a user’s specific intended hand motion	Is able to differentiate specific hand motions and does not require electrode placement by the patient	Cannot be used in patients with complete hand paralysis
Pressure Sensors - Digits			
Bend Sensors – Wrist	A bend sensor is placed on the wrist as wrist motion is likely still a familiar motion in patients with hand impairment	Simple to implement and can reliably detect wrist motion. Does not require electrode placement by the patient	May not be able to distinguish specific hand motions and requires wrist motion to be intact
EEG	An EEG pattern analysis was obtained on healthy patients in order to be able to identify similar patterns in patients with hand paralysis	Can be implemented in a patient with complete paralysis because acquires signal for intent at the beginning of motor pathway	Requires many electrodes to be placed on the head and may be the least reliable means of detection of user intent of those presented
sEMG	Electrodes are placed on major muscles of the forearm to detect myoelectric activity in order to gauge user intent	Reliably detects forearm activity and is able to differentiate some specific hand motions	Requires some residual level of muscle activity
Voice activated	Voice commands can operate the device	Unambiguously controls the device	Not a part of neuromuscular pathway so effects on neuroplasticity are less clear

#### Human-robot interface

**Fig. 6:**
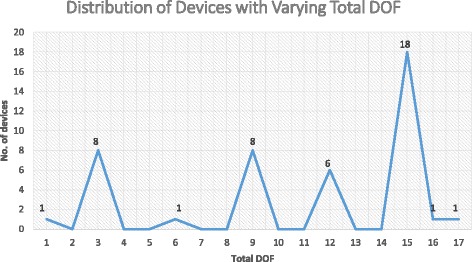
Distribution of devices with varying total DOF

A distribution of the total DOF per device in this review may be seen in Fig. 6. The most common approach was having 15 total DOF (18/44, 41%), which generally meant flexion/extension of all 5 digits. This enabled more functionality of the hand as every digit was actuated. The next most common trend was having either 3 or 9 total DOF (8/44 for each, 18%). Devices with 3 total DOF were those limited to a single digit and generally meant that the authors were bench testing their actuator design and that this was not the endpoint of their robotic device. On the other hand, devices with 9 total DOF were strategically designed to actuate only digits absolutely necessary to perform certain essential tasks, such as pinching or grasping. This helped reduce the weight of the device on the hand but its implications on rehabilitative ability was not immediately obvious. Finally, a handful of devices utilized 12 total DOF (6/44, 14%), which commonly meant all digits except the thumb were actuated in flexion/extension. This was often due to the thumb’s complex ROM which necessitated a separate, unique actuator. Given the thumb’s importance in performing routine actions such as grasping, we expect more devices to begin incorporating the thumb in their devices. In addition to total DOF, we believe that the number of independent actuators was worth including in the analysis as the unique combinations and designs utilizing differing number of actuators was able to influence certain elements of the device’s performance. While the most common setups included using one actuator per digit or one actuator per entire device, there are a number of devices that used other unique combinations. For example, the Power Assist Glove by Toya et al. used one actuator to control the thumb, one actuator to control all the other MCP joints, one actuator to control both the DIP and PIP joints of the 2nd and 3rd digits, and a final actuator to control both the DIP and PIP joints of the 4th and 5th digits [18]. This helped distinguish specific actions of the hand, such as grasping vs pinching. Unfortunately, there were too many unique designs to succinctly summarize here so we refer our readers to Table 4 for a reference for those devices. Similarly, the unique approaches to the designs of the actuators themselves and their consequent bending dynamics are also worth observing but too difficult to summarize comprehensively here.

**Fig. 7:**
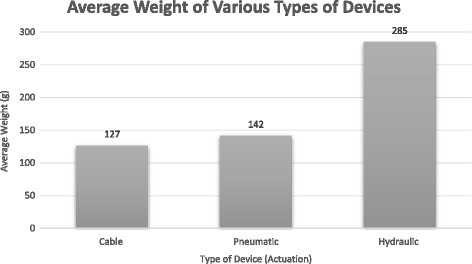
Average weight of different types of devices

In terms of the weight of the wearable portion of these devices, all devices with reported weights fell within the 0.5 kg requirement previously established. One exception included the RoboGlove, however, since this device was not designed as a rehabilitative device, it was excluded in this section. Groups that did not report a weight likely were early in development and still focusing on actuator design and did not have a wearable prototype of their systems yet. A comparison of the average weight of each of the categories of devices can be seen in Fig. 7. At this point, a statistical analysis of the groups would not yield significant results due to the small sample sizes. However, we expect to see a continuation of the current trend where the cable and pneumatic systems tend to be lighter than the hydraulic systems. This is likely due to the mechanical and structural demands of each type of actuation system and the materials needed to build them. Despite the minor trend, all these devices were well within the weight limit and so this was not perceived as an area of concern. Future work may also consider the size or profile of these devices and its impact on functionality. For now, future developers should continue to strive to reduce the weight of their devices as much as possible to reduce any unnecessary weight on the patient’s hand and promote a more ideal rehabilitative environment.

### Interaction with environment

#### Methods of evaluation

An index of the various metrics observed with brief descriptions is seen in Table 7. Because this section is interested in observing metrics that can be applied to all devices, results of experiments that were conducted to specifically evaluate a unique design element were excluded. Therefore, the reported list is composed of the previously used metrics that can either be applied to all these devices or to whole categories of devices (cable or pneumatic systems). In this analysis, we have included for comparison the most common metrics reported by devices: the input force and the grasp force (extension torque for extension devices). ROM was not reported consistently enough between devices and so was not included but we believe that it is a metric that is worth measuring because it helps understand if the devices are performing according to the anatomic ROM for each individual joint. In addition to these metrics, many experiments have also begun including motion trajectory analysis. An understanding of the motion path of the finger-robot tandem could provide a useful supplement to ROM measurements in quantifying whether correct anatomic movement is achieved. Another statistic, pinch force, was seldom reported in experiments because only a few devices were able to distinguish this motion. However, pinching is an essential part of performing ADL’s and so should be included when possible. Finally, speed or time of actuation was another metric that was beginning to become popular because devices that took too long to perform could lead to decreased patient satisfaction and compliance. Adherence to these commonly reported statistics should provide a method of comparison of these devices and allow a way to track the progression of the field of soft robotics in rehabilitation.

**Table 7:** Description of different metrics used by different devices

Measurement	Description
Extension torque	The torque applied by the device on finger extension
Grasping ability	Tested whether subject was able to grab various objects with assistance from the device
Grip force	The force exerted by the device attempting a grasping motion with subject completely passive
Max input force	Either the max input force supported by the device or the max input force required to achieve the desired functionality (pneumatic and hydraulic systems only)
Motion trajectory	Tracks the trajectory of the device/digits upon actuation
Opposition grasp force	The actuated force achieved while opposing the thumb
Pinch force	The force exerted by the device attempting a pinching motion with subject completely passive
ROM	Measurement of the rotations about the joints in the hands
Speed of movement	Speed of movement of the fingertip upon actuation
Tensile force	The max tension required to achieve desired function (cable systems only). It is the equivalent to max input force of pneumatic systems.

#### Modes of rehabilitation

Many of the devices used in this analysis were developed primarily as assistive devices or still early in development as rehabilitative devices and there was little quantitative data to report. However, there is still a general trend in the direction of modes of rehabilitation these devices intend to implement. Currently, Task Specific Training (TST) was the most common form discussed as it aims to allow patients to begin recovering function in their hand. TST is often one of the first rehabilitation exercises and is likely to continue to be a staple of robotic rehabilitation. The next most common training modality was Continuous Passive Motion (CPM), which is likely to continue to rise in popularity because of its ease of implementation and functional efficacy. CPM is focused on improving range of motion and recovering strength, a logical next step in the rehabilitative process. Finally, Active Resistance (AR) was the least common exercise implemented, probably because it represents one of the final stages of rehabilitation: strength training. As more devices progress to their evaluation phases, AR exercises are expected to increase in popularity. Ideally, these robotic devices should incorporate all of these exercises in order to have a more complete rehabilitation regimen. This need for an all inclusive rehabilitation program has likely partially inspired the development of Virtual Reality (VR) systems [19, 20], which can include many of these types of exercises. The major benefit for VR rehabilitation is that it engages the patient more, which should lead to more active involvement and better results. While there were only two devices reported to have used VR, it is an area of research that is commanding more attention as more information about its efficacy is surfacing.

## Conclusion

Since the emergence of soft robotic devices for hand rehabilitation roughly 10 years ago, the field has progressed rapidly. Significant progress has been made in establishing proof-of-concept of designs of preclinical research prototypes, with clinical trials being the next logical goal. But for many devices, more work needs to be done to perfect actuator design and feedback to maximize patient safety and rehabilitation outcomes.

A framework was developed for comparison of these devices based on critical features of the devices and the availability of these details in the literature. Beginning with the *Control Unit*, we have seen only a number of devices beginning to consider portability in mind, but we expect this to steadily increase as the field advances due to the perceived benefits of at home rehabilitation. Likewise, safety did not appear to be a major concern for many devices, but as more devices move to testing on human subjects, we expect it to become more relevant. This analysis may serve as a reference for those looking to incorporate safety features (or any other elements of our framework) into their devices. We hope that it may also serve as a guide for the different feedback mechanisms that have been implemented, which are gaining ground as ways of facilitating rehabilitation that are unique to robotics. On our conceptualized *Wearable Orthosis*, we have noticed a trend towards the production of Pneumatic Systems as well as devices that strive for increased total DOF and number of independent actuators. For the latter, this may allow for the ability to assist specific hand motions. This section also included a brief investigation of the weight of these devices, which were well within the limits for all devices presented. Additionally, our framework includes a discussion of the devices’ *Interaction with Environment*. This includes an index of some of the more common or more relevant metrics that have been used to evaluate these devices, which we hope may guide future experimentation and facilitate an ease of comparison between the devices’ functionality. We also include a brief discussion on the roles of the different modes of rehabilitation that have been considered in these devices, which we hope future developers will consider as they move onto clinical trials.

The list of design criteria that was analyzed is far from exhaustive in terms of all of the considerations needed. Nevertheless, different solutions to developing these devices were presented by highlighting key design features as well as some of their advantages and disadvantages. We hope that this review of the current approaches in designing soft robotic devices for hand rehabilitation will serve as a useful resource for future developers and facilitate the evolution of the field.

## Abbreviations

ADL: Activities of daily living
AR: Active resistance
CPM: Continuous passive motion
DIP: Distal interphalangeal
EEG: Electroencephalography
MCP: Metacarpophalangeal
PIP: Proximal interphalangeal
ROM: Range of motion
sEMG: Surface electromyography
TST: Task specific training
VR: Virtual reality
